# Compliance with Veterinary Advice on Dog Walking Is Influenced by Owners’ Beliefs

**DOI:** 10.3390/ani16192975

**Published:** 2026-09-22

**Authors:** Bronwen E. Bollaert, Daniel S. Mills, Jacqui M. Ley, Helen E. Zulch

**Affiliations:** 1 Animal Behaviour, Cognition & Welfare Group, Department of Life Sciences, University of Lincoln, Lincoln LN6 7DL, UK; bbollaert@lincoln.ac.uk or info@healthypetbehaviourvet.com.au (B.E.B.); dmills@lincoln.ac.uk (D.S.M.); 2 Healthy Pet Behaviour Services, Brisbane, QLD 4034, Australia; 3 Melbourne Veterinary Specialist Centre, Glen Waverley, VIC 3150, Australia; drjley2013@gmail.com

**Keywords:** veterinary advice, client compliance, behavioural problems, dog walking, owner beliefs

## Abstract

Dog owners may not always comply with veterinary advice on walking their dogs. This study surveyed dog owners internationally to elucidate factors which may be driving owner decision-making and variations in compliance. Four hypothetical scenarios were presented where the veterinarian advised the owner to walk their dog less for a medical (arthritis/degenerative joint disease) or behavioural (fear/aggression to something commonly encountered on a walk) reason, or more for a medical (overweight) or behavioural (improving mental well-being) reason. Owners were significantly less likely to comply with veterinary advice to walk their dog less for a behavioural reason. Multivariate analysis produced a minimum adequate model with four significant variables to explain differences in compliance with advice for walking less for a medical versus a behavioural reason: I do NOT feel that my vet expects me to walk my dog; I believe that walking my dog is important for my dog’s physical health; Walking my dog gives me important “quality time” in nature; I do NOT feel that walking my dog is important for my dog’s social interactions with people. Owners’ beliefs around these factors were most influential in the prioritisation of physical over behavioural health. Awareness of owner beliefs, particularly those involving walking less for behavioural reasons, may enable veterinary teams to use appropriate counselling tools to bring about improvements in outcomes for patients.

## Simple Summary

Dog owners may not always comply with veterinary advice on walking their dogs. This survey presented owners with four hypothetical scenarios where the veterinarian advised them to walk their dog less for a medical (arthritis/degenerative joint disease) or behavioural (fear/aggression to something commonly encountered on a walk) reason, or more for a medical (overweight) or behavioural (improving mental well-being) reason. Owners were significantly less likely to comply with veterinary advice to walk less for a behavioural reason than for the other scenarios. Four attitude statements were the best predictors of when owners prioritised physical over behavioural health: I do NOT feel that my vet expects me to walk my dog; I believe that walking my dog is important for my dog’s physical health; Walking my dog gives me important “quality time” in nature; I do NOT feel that walking my dog is important for my dog’s social interactions with people. Understanding owners’ beliefs around these factors is important to appreciate the risk of non-compliance with the advice being given by veterinarians. For those working with behavioural issues and advising reduced exercise as part of a behaviour management programme, it is important to be aware of these issues and to use appropriate counselling to maximise compliance.

## 1. Introduction

While walking pet dogs has been associated with benefits for human mental, physical and social wellbeing [1,2,3], there is very little research examining the benefit of dog walking for dogs. This presents a challenge to veterinarians in providing sound clinical advice, backed by appropriate studies, to dog owners about appropriate walking and exercise for dogs in general, and individual patients in particular. This is despite Rohlf and colleagues’ (2012) finding that nearly all respondents (dog owners) to their survey believed that regular exercise is good for a dog’s behaviour (99.1%), promotes the relationship between an owner and their dog (99.9%) and maintains ideal weight and body condition (99.1%) [4]. Several factors have been found to influence the amount dogs are walked; for example, Westgarth et al. (2015) found that the number of dogs owned (“multiple”) and the number of people in the household (“more people”) were each negatively associated with daily walking, whereas there was a positive association with the size of the dog (medium and possibly large dogs) [5]. In addition, a number of factors relating to the dog–owner relationship and the dog’s behaviour were also associated with daily walking: having acquired the dog for a hobby, and the dog lying on furniture were positively associated; whereas the dog lying on laps, growling at household members, and playing chase games with the dog, were all negatively associated [5]. To date, the research on the impact of walking on dogs has tended to focus on issues such as how, under different conditions, dogs interact with their environment and social stimuli on walks. For example, Westgarth and colleagues (2010) examined the nature and frequency of interactions of pet dogs with other dogs, people and the environment whilst being walked, and assessed the effect of the use of a leash in modulating such interactions [6]. Foltin and Ganslosser’s study (2021) found that dogs walked off-lead in a wooded environment showed great variation in exploratory patterns of locomotion and travelled further than their owners [7]. Male dogs tended to spend more time and greater distances away from their owners than females did [7]. Potential reasons discussed for these sex differences included females reportedly being more social with humans in their interspecific interactions, hence being more likely to stay closer to their human companions, and males as being bolder, more proactive and explorative, hence being more likely to explore at greater distances [7]. Other authors have focused on the value of walking for specific subpopulations of dogs; for example, Gunter et al. (2017) examined the impact of encouraging dog walking on returns of newly adopted dogs to a shelter and found that participation in dog walking did not reduce return rates [8]. They also reported that the guardians’ sense of obligation, dog-walking self-efficacy, and perceptions about their dogs’ on-leash behaviours were also not predictive. Despite the widely held belief by the general public (dog owners in particular), and professionals (veterinary and allied), that walking dogs is beneficial for dogs’ physical and behavioural health [7,9,10], there appears to be no published research specifically focused on the value of walking for dogs, or the relative value of different walking regimens. Without specific studies, veterinarians may struggle to provide scientifically grounded advice regarding appropriate exercise for their patients with their particular physical or behavioural needs. While a veterinarian’s role is to advocate for patient welfare, advising owners to modify walking routines can be difficult—especially when owners believe these routines are essential for their dog’s physical and behavioural health, and when the general literature cannot be called upon for explanation and reasoning.

Exercise and walking regimes may be modified for several reasons. Marcellin-Little et al. (2005) investigated the rehabilitation and conditioning of sporting dogs and concluded that further research needs to be conducted to determine the optimal amount of exercise needed for both non-sporting and sporting dogs [11]. Veterinary treatment plans and the management of behavioural issues may also require modifications to walking routines. Medical reasons include rehabilitation after orthopaedic procedures [12], injury prevention, and the management of osteoarthritis [13], as well as the management of overweight and obese dogs [14]. Common behavioural reasons for modifying walks for dogs include impaired social functioning [15] and reactivity when on-leash [16], since avoiding situations of high stress and reducing the risk of rehearsal of problem behaviours are fundamental aspects of a treatment plan. As a result, walking less or modifying how dogs are walked may be required [15,16,17,18]. Veterinary behaviour clinicians sometimes find it challenging to understand why clients are reluctant to implement advice of this nature, even when the reasons for doing so have been explained, and the client verbally confirms their understanding. If owners cannot implement and comply with veterinary or behaviourist advice, it is reasonable to expect that progress towards behavioural improvement will be limited. The factors influencing why owners may not comply with advice to reduce dog walking for behavioural issues, and the overall level of compliance with these recommendations, are still largely unknown.

Compliance has been defined within the medical field as the extent to which a human patient’s behaviour (whether taking medications, following dietary advice, or making other lifestyle changes) coincides with medical or health advice [19]. In the veterinary field, client compliance is the extent to which a client is able to implement the veterinarian’s advice (medical, behavioural or otherwise health-related). Several studies have investigated factors that may influence client trust, satisfaction and thus compliance/adherence with the advice given (see Lamb et al. 2018 for a review) [20]. For example, veterinarian communication skills [21], and dog owners’ knowledge of primary health care, their interest in learning about it, their opinion on it, perceptions of their veterinarian’s role regarding primary health care and satisfaction with their veterinarian regarding primary health care, were all factors affecting trust in veterinary advice and satisfaction with that advice [22]. Thus, the clinician–client relationship is clearly important and veterinarians using a relationship-centred care approach (characterised as a collaborative partnership between a veterinarian and a client, the provision of clear recommendations, and effective communication of the rationale for the recommendations) achieve greater client adherence to the advice they offer [23]. Whilst providing clear recommendations in a relationship-centred approach are likely to improve compliance, they cannot guarantee it. Understanding other factors which affect owner decision-making and subsequent behaviours relating to their pets will undoubtedly help to improve compliance. In the human field, it is well-recognised that changing behaviours can be challenging, and finding effective strategies to bring about change can also be difficult, even when the benefits are evident [24]. Understanding ways in which owners’ actions and behaviours are influenced allows veterinary clinicians to begin to address obstacles which impair implementation of their advice.

The Theory of Planned Behavior/Reasoned Action (Ajzen 1985; Ajzen 1991) [25,26], has emerged as one of the most influential and popular conceptual frameworks for the study of human action (Ajzen 2002) [27]. The theory aims to predict volitional human behaviours and improve understanding of their psychological determinants. It states that human decision-making and behaviour are affected by three factors: the individual’s intentions, their attitudes (beliefs about a behaviour), and subjective norms (beliefs about others’ attitudes toward a behaviour). An individual’s perceived behavioural control, self-efficacy and locus of control (beliefs about their control over a particular behaviour) also form part of this theory and play a role in predicting goal-directed behaviours. These factors may play a role in owners’ beliefs about how and why they walk their dogs, whether their behaviours are influenced by others around them, and their perceptions of what is expected of them as dog owners more generally. Understanding their sense of control over modifying a behaviour gives additional nuance to whether they may or may not implement changes advised by their veterinarian.

Thus, the aim of this study was to investigate why owners may not always comply with veterinary advice, and what influences their decision-making with respect to dog walking. We hypothesised that owner decisions regarding dog walking are influenced by more than the dogs’ behaviour or physical needs, or the veterinarian’s recommendations, and so undertook a survey to evaluate what other factors may be important.

## 2. Materials and Methods

### 2.1. Survey and Data Collection

An online survey (see Supplementary Survey S1), hosted on JISC (20 August 2024—31 January 2025) was distributed internationally using snowball sampling through emails to veterinary colleagues, social media platforms, and recruitment posters displayed in veterinary clinics. Participants were required to currently own a dog, be at least 18 years of age, have internet access, and provide their consent to participate. Participation was voluntary and anonymous. Participants did not receive any remuneration and were able to opt out at any time. For the purposes of completing the questionnaire, they were asked to respond in relation to their current dog; if they currently owned more than one dog, they were to focus on the dog whose name came first alphabetically.

Participant demographic data was collected including the participant’s age and gender, the number of human household members, and their ages. Information about the home included the country in which the participant lived, the total number of dogs in the household, whether the dog had access to an outdoor area for toileting and relaxation, and the location of the home (city centre, suburb, village, rural, etc.). Dog demographic data included age of the dog, sex and neuter status, breed, size/weight and whether the participant currently walked their dog or not.

Participants were then asked to consider factors that have influenced their decisions about walking their dog. Ajzen’s Theory of Planned Behavior/Reasoned Action [25,26,27] was considered when designing the questionnaire statements. Factors included what participants believed their dog’s veterinarian, their dog’s trainer, any significant other/friends/family members, and their communities thought about dog owners walking their dogs, and how their own beliefs may influence how or whether they walked their dogs. Participants rated their agreement with each statement using 5-point Likert scales, which were later coded (1 = Strongly Disagree; 2 = Disagree; 3 = Neither Agree nor Disagree; 4 = Agree; 5 = Strongly Agree).

Finally, four hypothetical scenarios were presented in which a veterinarian had made a diagnosis of a condition in their dog and advised on the modification of their dog’s walks as a result. These were as follows: walking less frequently/shorter distances following a diagnosis of arthritis/degenerative joint disease (walking less for a medical reason); walking less following a diagnosis of fear aggression to something commonly encountered on walks (walking less for a behavioural reason); being advised the dog was overweight and needed to walk more often and for longer duration (walking more for a medical reason); and being advised that in order to improve mental well-being they were to walk more often/for a longer duration (walking more for a behavioural reason). Questions were aimed at uncovering some of the owners’ perceptions of whether they would trust this advice given the assessment, how easy or difficult it would be to implement the advice, and other factors that may influence their implementation of this advice. Participants rated their likelihood of compliance using 5-point Likert scales, which were later coded (1 = Strongly Disagree; 2 = Disagree; 3 = Neither Agree nor Disagree; 4 = Agree; 5 = Strongly Agree).

### 2.2. Data Analysis

In order to control for response consistency, those participants that responded differently for the statements “I would find it difficult to comply as this is the way my dog goes to the toilet” and “I would find it difficult to comply as it’s the only time my dog gets outside” within the two scenarios relating to walking less for a medical and walking less for a behavioural reason were removed.

Data were analysed using IBM SPSS (v29). Descriptive summaries were created, before the significance of differences in compliance between the four scenarios was assessed initially using bivariate analyses using a Friedman test.

After this, a new dependent variable (medvsbehavpriority) was then created based on the difference in likelihood to comply with a reduced exercise recommendation for a medical versus behavioural reason (medical rating minus behavioural rating). A positive value indicated a bias towards compliance for medical reasons, while a negative value indicated a bias towards compliance for behavioural reasons; zero indicated no bias. Bivariate associations between this variable and ratings of the statements relating to factors affecting their dog-walking behaviour were then evaluated. This identified potential factors of importance in predicting a bias towards prioritising physical over behavioural health. However, in order to identify the most important factors, we then used the significant factors in a series of generalised linear models. To reduce the risk of issues from the overspecification of variables, the agreement-rating scales were reclassified into a three-point scale (strongly agree and agree merged into a single category, and the same done for the two levels of disagreement). A backwards model-building procedure was then used to produce the Minimum Adequate Model (MAM). Model building stopped when there were only significant variables remaining in the model, with the Akaike Information Criterion (AIC) used to assess model adequacy at each stage.

Given the results from the initial bivariate analysis, an equivalent analysis was not undertaken for explaining the likelihood of increasing exercise in relation to a medical versus behavioural reason.

Simple bivariate analyses were used in the first instance, with an uncorrected significance threshold alpha set at *p*  =  0.05, since in this type of exploratory discovery work the impact of a type 2 statistical error is of more concern than the impact of a type 1 error in the consideration of potential factors worthy of further consideration (Perneger, 1998) [28]. The subsequent multivariate analysis sought to find the best explanatory variables, and so provides further insight into the best predictors, but not necessarily the most biologically relevant factors.

## 3. Results

There were 1590 complete responses submitted. After applying the filter based on consistency relating to toilet and outside (see above), 1098 responses were retained. All analysis was performed on this data set.

The majority of participants were women, and nearly half (47.4%) of the participants were between the age of 35 and 54 years old. Participants were from the following countries, in descending order of frequency: United States of America (37.8%), Australia (25.1%), the United Kingdom (20.9%) and the remaining 16.2% from other countries. The majority of participants live in suburban areas (53%). Full details of owner demographics can be found in Table 1.

The majority of participants’ dogs were reported as being between 2 and 7 years old (58.8%), 47% were females and 53% were males, and the vast majority were reportedly neutered (81%). The majority of dogs were reported as being large and giant breeds over 21 kg (54.9%) with medium-sized dogs (11–20 kg) at 29.8% and toy/miniature and small dogs (<10 kg) comprising 15.3% of the population. The majority of dogs were reported to have access to small- to moderate-sized (46.1%) and extensive (40.5%) outdoor spaces, with the remainder having access to a very small outdoor space (5.1%) or access only under active supervision (8.3%). Respondents reported that 92% of the dogs were currently walked. Full details of dog demographics can be found in Table 2.

For the statement “I would comply with all the advice as my vet knows what is best for my dog’s health”, 85% of owners indicated that they were likely to comply with veterinary advice to walk their dog less frequently/for shorter distances following a hypothetical diagnosis of arthritis/degenerative joint disease, compared with 63% for demonstrating aggression due to fear of something commonly encountered on a walk. For the scenarios where owners were advised to walk more/for a longer duration following advice based on their dog being hypothetically overweight or for mental wellbeing, 82% agreed or strongly agreed to comply with veterinary advice for medical versus 79% for behavioural reasons.

Owners were significantly less likely to comply with veterinary advice to walk their dog less for a behavioural reason than for a medical reason (Q = 0.659, Std test stat 11.959, *p* < 0.001); and significantly less likely to comply with advice to walk less for a behavioural reason than to walk more for a medical (Q = −0.482, Std test stat −8.752, *p* < 0.001) or more for a behavioural reason (Q = −0.383, Std test stat −6.959 *p* < 0.001). They were also significantly more likely to comply with advice to walk their dog less for a medical reason than to walk their dog more for a behavioural reason (Q = 0.276, Std test stat 5.000 *p* < 0.001), and more for a medical reason (Q = 0.177, Std test stat 3.207, *p* = 0.01). There was no statistical difference in the likelihood of them complying with advice to walk their dog more for a medical versus a behavioural reason (Q = 0.099, Std test stat 1.793, *p* = 0.437) (see Supplementary Table S1 for these results). These results indicate that owners were least likely to comply with advice to walk less for a behavioural reason, and most likely to comply with advice to walk less for a medical reason.

The following variables were found to be significant in the bivariate analysis and were considered for inclusion in the MAM: ‘I do not feel that my vet expects me to walk my dog’; ‘Walking is integral to how my dog goes to the toilet’; ‘Walking my dog is an important part of my daily routine’; ‘Walking is the only time my dog gets outside’; ‘I have easy access to dog friendly walking tracks and paths for walking my dog’; ‘I feel that my dog gets excited to go for walks’; ‘I believe that walking my dog is important for my dog’s physical health’; ‘I feel that walking my dog is important for my own physical health’; ‘I feel that walking my dog is important for me to see friends/family/neighbours’; ‘I feel that members of my family or a “significant other” thinks that walking a dog is the responsible thing to do as a dog owner’; ‘I feel that the community I live in expects dogs to be walked regularly’; ‘I feel that walking with my dog is important for my own mental well-being’; ‘Walking is not a relaxing activity for me’; ‘I do not feel that walking my dog is important for my dog’s social interactions with people’; ‘Walking my dog gives me important “quality time” in nature’; ‘Walking gives my dog important “quality time” in nature’; ‘I feel that walking my dog is expected of me as a responsible dog owner’; ‘I do not look forward to going for walks with my dog’; ‘I feel that family commitments make it difficult to walk my dog’; ‘I believe that walking my dog is important for my dog’s social interactions with other dogs’ (see Supplementary Table S2).

For the multivariate analysis, AIC consistently dropped at each stage until a final model remained with four significant explanatory variables (AIC 289.5): I do NOT feel that my vet expects me to walk my dog (Wald Chi sq 8.755, *p* = 0.013); I believe that walking my dog is important for my dog’s physical health (6.721, *p* = 0.035); Walking my dog gives me important “quality time” in nature (35.007, *p* < 0.001); I do NOT feel that walking my dog is important for my dog’s social interactions with people (124.354, *p* < 0.001).

## 4. Discussion

The aim of this study was to understand more about why dog owners may not always comply with veterinary advice with respect to managing their dog’s behaviour, and what influences their decision-making with respect to complying with advice about dog walking, in particular. The authors hypothesised that owner decisions regarding dog walking are influenced by multiple factors, and not necessarily their dogs’ behaviour or physical needs, or their veterinarian’s recommendations.

Our study found that dog owners’ compliance with veterinary advice varied across the four hypothetical scenarios. For the statement “I would comply with all the advice as my vet knows what is best for my dog’s health”: 85% agreed or strongly agreed with walking less for a medical reason, 82% agreed or strongly agreed for walking more with a medical reason, and 79% agreed or strongly agreed with walking more for a behavioural reasons, but only 63% agreed or strongly agreed with walking less for a behavioural reason. Owners were significantly more likely to comply with advice to walk less after their dog received a diagnosis of arthritis/degenerative joint disease; as likely to comply with veterinary advice to walk more for a medical or behavioural reason; and significantly less likely to comply with advice to walk less for a behavioural reason. This indicates that owners surveyed in this study displayed a bias towards prioritising physical health over behavioural health. The MAM identified four factors that may provide insight into some of the influences on owners’ decision-making when they make choices contrary to advice given by their veterinarian: owners’ beliefs about what is important for their dog’s physical health, how they viewed their veterinarian’s expectations about walking their dog, their own need for quality time in nature, and their dog’s need for social interactions with people.

Our findings are supported by the experience of behaviour clinicians who anecdotally report that clients seem to struggle with implementing advice to reduce walking as part of a behaviour treatment plan. Dog owners reported that they may not always comply with veterinary advice on dog walking even after a diagnosis is made and specific modifications are advised as part of a treatment plan. The reasons that they give for this are multifactorial and need to be understood, as walking modifications may be an essential component of an effective behaviour treatment plan [15,16,17,18]. If owners are not able to implement these strategies and comply with veterinary or behaviourist advice, it is reasonable to expect that progress towards behavioural improvement will be limited.

Adherence to veterinary advice is influenced by a number of factors [29,30]. Some of these are within the clinician’s control such as providing written instructions and visual aids, adjusting therapy according to the patient’s response, and sending reminders for follow-up appointments [26]. However, they may have less control over others, particularly those affecting an individual’s decision-making and behaviour. These include clients’ intentions, attitudes, subjective norms, perceived behavioural control, self-efficacy, and locus of control regarding a particular behaviour [25,26,27]. Understanding these factors will enable the clinician to address and remove potential barriers to the implementation of advice. In the medical field, clinicians (especially those involved with behavioural change) are attempting to move towards the concept of concordance with advice [31]. Concordance emphasises an agreement between the clinician and the patient; it considers each other’s perspective on a particular treatment; and it includes open discussions on recommended treatment steps, information sharing and supporting patients, which is especially important for those on long-term treatment plans [31]. It is a process that acknowledges that patients’ views have to be respected even if they make choices that appear to be in conflict with the clinician’s views [31]. Shifting veterinary clinicians towards a perspective of concordance with clients may assist in developing treatment plans that improve treatment outcomes for veterinary patients. Thus, an awareness of reasons such as those found in this study can improve the ability of primary care veterinarians, behaviour specialists, and others advising dog owners, developing concordant treatment plans together with clients.

Owners’ responses to ‘I believe that walking my dog is important for my dog’s physical health’ (only 51 of 1098 respondents disagreed or strongly disagreed) and to ‘I do NOT feel that my vet expects me to walk my dog’ (only 158 of 1098 respondents agreed or strongly agreed), and the finding that these factors remained in the MAM, indicate that these beliefs—about the importance of walking for their dogs’ physical health—are widely held by owners. Additionally, they suggest that the beliefs may be reinforced by their veterinarians. Other owner responses—walking for toileting needs, dog’s only time outside being during walks, being a single dog in the household (620 of 1098 respondents), a medium or larger dog (928 of 1098 respondents), walking providing their dog access to nature, were all significant in the bivariate analysis—relate to these key explanatory factors and appear to be intertwined with the owner’s beliefs about taking care of and attending to their dog’s physical needs.

In addition, when considering the factor ‘I do NOT feel that walking my dog is important for my dog’s social interactions with people’ (377 agreed or strongly agreed), the reverse of this statement, I do feel that walking my dog is important for my dog’s social interactions with people, indicates that the majority of owners also feel that walking their dog is a way of meeting these social needs for their dog. This means that there is an acknowledgement of the potential importance of walking for the dogs’ mental wellbeing as well as physical health. Positive affective experiences, expression of agency, and a variety of normal behaviours—including interactions with people, other animals and their environment—are recognised indicators of wellbeing and positive welfare [32]. All these can be met through appropriate walking regimes. Add to this the fact that when asked to consider ‘Walking my dog gives me important “quality time” in nature’, only 69 of 1098 disagreed or strongly disagreed. Owners may have the belief or have received advice from their own health care professional about dog walking being good for them [1,2,3]. This could immediately raise problems for the owner in trying to comply with advice for their own health to walk the dog more, conflicting with their vet’s advice to walk the dog less. Furthermore, if owners believe that walking the dog also meets their dog’s need for “quality time” in nature (only 35 of 1098 disagreed or strongly disagreed), it presents an even greater cognitive block. Rohlf and colleagues 2012 reported that owners agreed that walking was good for their dog’s behaviour, promoted their relationship, and maintained their dog’s ideal weight and body condition [4]. So, contrary to their vet’s advice to walk less, they may believe they should walk more to help their dog’s behaviour [7,8,10], especially in the absence of specific reasoning to the contrary.

Additionally, taking a dog for a walk could have been one of the primary reasons an owner adopted a dog in the first place. Holland and colleagues report that facilitating exercise was a frequent reason for wanting to get a dog, as expressed by 48.2% and 69.7% of current and potential dog owners, respectively [33]. If owners are advised not to undertake an activity with their dog that was one of the most important reasons for acquiring it, complying with the advice will clearly be difficult. Owners who do not walk their dogs may feel a sense of loss concerning the walks they thought they would have.

What owners are then navigating, when advised to walk their dogs less for a behavioural reason, is their belief that they may be compromising their own and their dog’s physical health, and their belief that their veterinarian expects them to walk their dog, potentially creating in an internal conflict with the advice that they are now being told not to do so. Added to this is the fact that veterinarians may not be comfortable fully exploring behavioural concerns with owners. Despite behavioural problems being a leading reason for euthanasia in shelter dogs and cats, they were not recognised as one of the top four welfare problems by 40% of small animal veterinarians in general practice [34]. Half of the respondents were reluctant to discuss behaviour problems with clients because of deficiencies in their behaviour knowledge, and 80% felt that their education equipped them poorly or extremely poorly to deal with behaviour problems in dogs and cats [34]. If qualified veterinarians feel ill-equipped to deal with behavioural concerns, this may go some way to explaining why behavioural problems may not be discussed or addressed in general practice frequently enough, and why they are a leading reason for euthanasia in shelters that may bear the consequences of these underdiagnosed and undertreated conditions. Considering that the general practice veterinarian is the most likely contact point for informed opinion and advice, if veterinarians generally feel they lack knowledge and skill in this area, disseminating appropriate behavioural knowledge and advice to dog owners will also be impaired. If behaviour problems are not routinely discussed with owners, then there may be a tendency for the owners to prioritise the importance of physical health concerns.

Veterinarians need to be able to discuss the inter-related nature of physical and behavioural health and understand owners’ beliefs and perspectives regarding these. These include the beliefs elucidated by the current study. Discussions need to include a compassionate and empathetic exploration of what walking the dog means for the owner’s physical and mental wellbeing and enable a discussion aimed at the veterinarian and the owner together, finding a solution that works for both the owner and the dog. The owner needs to be helped to see that they are not being faced with an ‘either/or’ decision, when it comes to physical versus behavioural health, but rather encourage a perspective that considers ‘what else could I do to fulfill my dog’s physical health needs that doesn’t compromise its behavioural health?’ Additionally, understanding the potential implications for the owner’s health and wellbeing is essential so that owner’s concerns regarding this can be addressed, as appropriate. If veterinarians identify underlying beliefs and can reframe them, they may assist owners in overcoming mental barriers and help identify other means to achieve the same ends, moving closer to compliance. This is an effective strategy that has been employed by a variety of professionals in several fields where problems are complex and require human behavioural change [35]. This approach may be facilitated by using counselling tools such as motivational interviewing to bring about better understanding, development and implementation of a personalised plan that the owner is involved in developing [36].

Further compounding this complex discussion around dog walking decisions, and impacting veterinarians’ ability to make evidence-based recommendations, is the fact that there is very little material published on the benefits or risks of dog walking on the dog. The authors are unaware of any publications that provide direct evidence-based recommendations for types of daily exercise—or on the frequency, intensity or duration of particular types of exercise—for any particular breed or age group of dogs, or for the physical health requirements for dogs in general. Additionally, the authors are unaware of any published studies that have investigated the benefits of walking for dogs’ behavioural health, despite the widely held belief that walking is good for the dog’s behaviour [7,9,10]. These are areas of research that need to be explored and would go a long way in supporting recommendations for treatments for veterinary patients.

There are limitations associated with surveys, including potential participation, self-selection and response biases which may apply to this study. Only 93 (8%) of the respondents reported not currently walking their dogs, and a large proportion of the dogs were large or giant breeds (54.9%). This may indicate that the survey has perhaps not reached a proportionate number of dog owners in the population that do not currently walk their dogs, or those who own smaller dogs. Whilst every effort was made to recruit a representative sample of the dog-owner population (both those who did and did not walk their dogs) through broad distribution channels and avoiding those targeting only a dog-walking population, some sampling bias may still remain. Controlling for dog size presents practical challenges since the popularity of certain breeds or sizes of dogs may vary substantially depending on the country and/or participant location. As far as the authors are aware, there is no worldwide database describing the current popularity of breeds or sizes of dogs which could clarify a current representative population proportion according to size. There are also factors such as the respondent’s personality (how likely they are to comply in general), the respondent’s previous experiences with veterinarians (positive or negative), the respondent’s experience of managing a dog with a behavioural problem, the respondent’s current veterinarian’s personality and communication style (authoritarian and forceful versus authoritative and collaborative) which could not be controlled for in this study, and may be valuable avenues for further investigation. Nonetheless, this study has allowed the collection of data from a large number of participants in several countries who live in a variety of environments and housing types. This indicates that these issues are not isolated to one particular location, but affect dog owners as a broad population. Additionally, the fact that certain influences emerged despite this variability suggests that they are strong factors within owner decision-making. The study offers a starting point for addressing these complex but clinically relevant factors when treating animal behavioural problems. Further objective research into client beliefs and compliance with veterinary advice after a dog has actually, rather than hypothetically, been diagnosed with a medical or behavioural problem is likely to provide additional clinical data and further clarify patterns of compliance.

## 5. Conclusions

The study provides important insight into the challenges involved in the practical implementation of and compliance with veterinary advice relating to a change in dog walking, particularly in the light of addressing behavioural problems. Improvements in under- and post-graduate training in veterinary behavioural medicine will facilitate behavioural problems being recognised, addressed and treated appropriately. This in turn will raise awareness within the veterinary field, as well as the general population, of the importance of addressing behavioural problems and their critical impact on animals’ welfare. More broadly, veterinarians need to be aware that dog owners’ underlying beliefs affect their decision-making and capacity to comply with treatment plans. These beliefs may be complex and intertwined. If clinicians are able to identify and address beliefs with a variety of counselling skills—for example, motivational interviewing and reframing—they may improve client–veterinarian relationships, their interpersonal communication, and client compliance with treatment recommendations, ultimately improving outcomes for dogs and their owners.

Applications of findings to veterinary clinical practice are as follows:

- Clients have underlying beliefs which may affect their ability to comply with veterinary treatment plans;
- Veterinarians need to be aware of underlying beliefs affecting compliance to address them;
- Owners may find it especially difficult to comply with veterinary treatment plans for a behavioural problem that specifies that an owner should walk their dog less;
- Veterinarians need to develop additional skills in veterinary behavioural medicine and counselling to identify problems and develop treatment plans that take into account client input and motivation;
- Suggested counselling skills include reframing and motivational interviewing;
- Future work may explore owner beliefs more specifically and the development of evidenced-based standard recommendations for dog exercise and walking, as well as modifications for age, breed and medical conditions.

## Figures and Tables

**Table 1:** Demographics of participants.

Gender of Participants: (Actual Numbers n = 1098)						
Women	Men	Non-binary		Preferred not to answer		Self-described
93.0% (1021)	4.3% (47)	1.8% (20)		0.8% (9)		0.1% (1)
**Ages of participants: (actual numbers)**						
<35 years		35–54 years		>55 years		
30.8% (338)		47.4% (521)		21.8% (239)		
**Participant country of residence: (actual numbers)**						
USA	Australia	United Kingdom	Canada	European countries		Rest of world
37.8% (415)	25.1% (276)	20.9% (229)	6.2% (68)	5.5% (60)		4.5% (50)
**Participant home location:**						
Suburban household	Inner city suburb	Village	Rural/farm/national park/reserve	Small holding/acreage	Town centre	City centre
53% (581)	11% (119)	12% (132)	8% (87)	7% (77)	5% (56)	4% (46)

**Table 2:** Demographics of dogs owned by participants.

Dog Age: (Actual Numbers n = 1098)			
<2 years old	2–7 years old	8–13 years old	14+ years old
17.9% (196)	58.8% (646)	20.8% (228)	2.5% (28)
**Sex of dog: (actual numbers)**			
Female		Male	
47% (513)		53% (585)	
**Dog neuter status: (actual numbers)**			
Neutered/desexed		Entire	
81% (890)		19% (208)	
**Dog size: (actual numbers)**			
Toy/miniature & small (1–10 kg)		Medium (11–20 kg)	Large & giant (21+ kg)
15.3% (168)		29.8% (327)	54.9% (603)
**Dog access to outdoor space: (actual numbers)**			
Extensive area	Small-moderate	Very small	Only under active supervision
40.5% (445)	46.1% (506)	5.1% (56)	8.3% (91)
**Current walking activity: (actual numbers)**			
Dogs that are currently walked		Dogs that are not currently walked	
91.5% (1005)		8.5% (93)	

## Data Availability

Data are available from the authors upon reasonable request.

## Supplementary Materials

The following supporting information can be downloaded at: https://www.mdpi.com/article/10.3390/ani16192975/s1, Survey S1: Decision making around dog walking; Table S1: Results of Friedmans pairwise comparison tests for “I would comply with all the advice as my vet knows what is best for my dog’s health” for walking more and less for behavioural and medical reasons; Table S2: Bivariate Analysis of medvsbehavpriority to determine where owners who prioritised medical over behavioural differed.
