# Supplementary material for: Compliance with Veterinary Advice on Dog Walking Is Influenced by Owners’ Beliefs
Source: Animals (Basel). 2026 Sep 22;16(19):2975. doi: 10.3390/ani16192975 (PMC13663331; doi:10.3390/ani16192975)
Supplement: Supplementary file 1 [file animals-16-02975-s001.zip › animals-4544935-supplementary.pdf]

Supplementary Material for: Compliance with veterinary advice on dog walking is influenced by owners' beliefs

S1: Survey: Decision making around dog walking

JISC Survey Site with Survey Logic

Survey Questions:

Instruction: Please complete the survey with reference to your own dog. If you own more than 1 dog, the survey is to be completed with only 1 dog in mind. Please select the dog whose name comes first alphabetically and complete the survey for this dog.

The survey will take approximately 15 minutes to complete and should only be completed once.

Consent to Participate in Research:

1) I confirm that I have read the information sheet dated 4th August 2024 for the above study. I have had the opportunity to consider the information, ask questions and have had these answered satisfactorily - Confirm (required to proceed)
2) I understand that as I have completed the study anonymously it will not be possible to remove any information I have provided, as you will not be able to identify me in any way. – Confirm (required to proceed)
3) I understand that individuals from the University of Lincoln may look at research data collected during the study, to ensure that the study is conducted appropriately. – Confirm (required to proceed)
4) I understand that the information collected about me will be used to support other research in the future and may be shared anonymously with other researchers. – Confirm (required to proceed)
5) I agree to take part in the above study. – Confirm (required to proceed)

Section 1: Owner demographics:

6) Age: <18 (END SURVEY); 18-24, 25-34, 35-44, 45-54, 55-64, 65-74, 75+
7) Gender: Woman, Man, Nonbinary, prefer not to answer, prefer to self-describe
8) Prefer to self-describe: – free text response
9) Number of adults (people 18 years older or more) in household:_____
10) Number of children under 3 years old in the household: ____
11) Number of children between 3 – 12 years old in the household: ___
12) Number of children between 13 – 17 years old in the household: ____
13) Total number of dogs in the household: ____
14) Housing: Does your dog have access to an outdoor area where it can toilet and spend time? ***Drop down menu (select one):*** Home with no associated outdoor area; home with associated communal area (e.g. garden in the complex); home with balcony / courtyard accessible to your dog; home with small to moderate garden / yard accessible to your dog; home with extensive garden / yard / outdoor area accessible to your dog; Other: ________ (free text response)
15) Housing: Other: ________ (free text response)
16) Please indicate which of the following best describes the location of your home: ***Dropdown menu (select one)*** City centre; Town centre; inner city suburb; suburb; village; small holding / acreage; rural / farm; nature reserve / national park; other ___
17) Other: ________ (free text response)
18) Country of residence: ***Drop Down Menu (select one):*** https://www.britannica.com/topic/list-of-countries-1993160

Section 2: Dog Demographics:

Instruction: Please complete the survey in reference to your own dog. If you own more than 1 dog, the survey is to be completed with only 1 dog in mind. Please select the dog whose name comes first alphabetically.

19) Current Age: ***Dropdown List (select one):*** 0-6 months old; 7-12 months old; 13-18 months old; 19-23 months old; 2-3 years old; 4-5 years old; 6-7 years old; 8-9 years old; 10-13 years old; 14-16 years old; >16years
20) Sex: ***Dropdown list one option only***:
    Male or Female
21) Neuter Status: Please select one option only ***Dropdown list one option only***:
    Desexed / Spayed / Neutered or Entire/ Intact or Unsure
22) Breed - ***Drop Down and Other:*** ______
23) Other breed description: ________ (free text response)
24) Size of dog (weight): ***Dropdown list one option only***:
    Toy / Miniature (1-5kg / 2.2-11 lbs); Small (6-10kg / 12 – 22 lbs); Medium (11 – 20kg / 23 – 44 lbs); Large (21 - 40kg / 45 – 88 lbs); Giant (Over 40kg / over 88 lbs)
25) Do you currently walk your dog? ***Dropdown list one option only***: Yes / No

Section 3: Factors that might influence dog owners' decisions about dog walking:

Whether or not you currently walk your dog, consider the factors that have influenced your decisions in the past or you believe may do so in the future. Please select one option for each statement.

Likert Scale options for all: Strongly agree; Agree; Neither Agree nor Disagree; Disagree; Strongly Disagree; Not applicable

26) I do not feel that my vet expects me to walk my dog
27) I feel that my dog trainer expects me to walk my dog
28) Walking is integral to how my dog goes to the toilet
29) Walking my dog is an important part of my daily routine
30) The weather is not a significant influence on how, when and/or where I walk my dog
31) Walking is the only time my dog gets outside
32) My dog's behaviour has a big influence on how, when and/or where I walk
33) My dog's physical health affects how, when and /or where I walk
34) I do NOT feel that my dog needs as much walking when my dog participates in certain other activities (e.g. dog sports)
35) My dog has access to an enclosed area (e.g. garden / dog park) and therefore I do not feel that my dog needs to go for walks
36) Walking is important "quality time" my dog and I spend together
37) I have easy access to dog friendly walking tracks and paths for walking my dog
38) I believe that my community is supportive of me walking my dog in the local neighbourhood
39) I feel that my dog is difficult to manage at home if I don't take him / her for a walk
40) I feel that my dog gets excited to go for walks
41) I believe that walking my dog is important for my dog's physical health
42) I feel that walking my dog is important for my own physical health
43) I feel that walking my dog is important for me to see friends / family / neighbours
44) I feel that my family and/or friends think that it's good that I walk the dog

45) I feel that members of my family or a "significant other" thinks that walking a dog is the responsible thing to do as a dog owner
46) I feel that the community I live in expects dogs to be walked regularly
47) I feel that walking is important for my dog's mental well-being
48) I feel that walking with my dog is important for my own mental well-being
49) I believe that walking is a relaxing activity for my dog
50) Walking is not a relaxing activity for me
51) I do not feel that walking my dog is important for my dog's social interactions with people
52) I believe that my dog keeps me safe when out walking
53) Walking my dog gives me important "quality time" in nature
54) Walking gives my dog important "quality time" in nature
55) I feel that walking my dog is expected of me as a responsible dog owner
56) I do not look forward to going for walks with my dog
57) Other people's complaints / criticisms have affected how, when and/or where I walk my dog
58) Going for walks near my home is unsafe, so I have to plan how, when and/or where to walk my dog
59) My work schedule makes it difficult to walk my dog
60) I feel that family commitments make it difficult to walk my dog
61) I feel that one or more people in my community do not like dogs, so I find it difficult to walk near my home
62) My friends / family say that my dog should not be walked
63) Walking is my dog's only exercise
64) My vet has advised that I should modify where / when / how I should walk my dog
65) My dog trainer has advised that I should modify where / when / how I should walk my dog
66) I believe that walking my dog is important for my dog's social interactions with other dogs

Section 4: Hypothetical scenarios:

Instruction: Now we are going to present you with a number of fictious / imaginary scenarios involving your dog. Following this, we will ask you how much you agree with a series of related statements.

Scenario 1: Consider a hypothetical / imaginary scenario where your dog's veterinarian has diagnosed him / her with arthritis / degenerative joint disease and advised you to walk your dog less frequently or for shorter distances.

Please rate each of the following statements with regards to your response to this advice:

Likert Scale options for all: Strongly agree; Agree; Neither Agree nor Disagree; Disagree; Strongly Disagree; Not applicable

67) I would find it difficult as my dog really enjoys going for walks
68) I would feel guilty that my dog would not get the exercise that she / he needs
69) I feel I would find it difficult to meet my dog's mental well-being needs at home without walking her / him as much
70) I would miss the social interaction that I have with other people on walks
71) I would find it difficult to comply as my family and / or friends believe it's important for me to take my dog for walks
72) I feel it would be a relief that I would not have to keep walking my dog
73) I would find it difficult to comply as this is the way my dog goes to the toilet
74) I would find it difficult to comply as it's the only time my dog gets outside

75) I would find it difficult to comply as that's the way my dog and I spend time together
76) I would worry that she / he would gain weight if she /he walked less
77) I would find it difficult to comply as I feel my community believes walking a dog is essential
78) I would comply with all the advice as my vet knows what is best for my dog's health

Scenario 2: Consider a hypothetical / imaginary scenario where your dog's veterinarian has diagnosed him / her with fear aggression to something commonly encountered on walks and because of this has advised you to walk your dog less frequently.

Please rate each of the following statements with regards to your response to this advice:

Likert Scale options for all: Strongly agree; Agree; Neither Agree nor Disagree; Disagree; Strongly Disagree; Not applicable

79) I would find it difficult as my dog really enjoys going for walks
80) I would feel guilty that my dog would not get the exercise that she / he needs
81) I feel I would find it difficult to meet my dog's mental well-being needs at home without walking her / him as much
82) I would miss the social interaction that I have with other people on walks
83) I would find it difficult to comply as my family and / or friends believe it's important for me to take my dog for walks
84) I feel that it would be a relief that I would not have to keep walking my dog
85) I would find it difficult to comply as this is the way my dog goes to the toilet
86) I would find it difficult to comply as it's the only time my dog gets outside
87) I would find it difficult to comply as that's the way my dog and I spend time together
88) I would worry that she / he would gain weight if she / he walked less
89) I would find it difficult to comply as I feel my community believes walking a dog is essential
90) I would comply with all the advice as my vet knows what is best for my dog's health

Scenario 3: Consider a hypothetical / imaginary scenario where your dog's veterinarian has advised you that your dog is overweight and asked you to walk your dog more often and / or for longer duration.

Please rate each of the following statements with regards to your response to this advice.

Likert Scale options for all: Strongly agree; Agree; Neither Agree nor Disagree; Disagree; Strongly Disagree; Not applicable

91) I would fully comply as my dog really enjoys going for walks
92) I would feel guilty if I could not walk my dog more often than I currently walk it
93) I feel that I would find it easier to meet my dog's mental well-being needs by taking her / him out for walks more often
94) I would enjoy the social interaction that I would have with other people on walks
95) I feel my family and friends would support this as they think it's important for me to take my dog for walks
96) I feel that it would be a difficult to modify my schedule to walk my dog more
97) I would comply as this is the way my dog goes to the toilet
98) I would comply as it's the time my dog gets outside
99) I would comply as that's the way my dog and I spend time together
100) I would worry that she / he would lose too much weight if I walked more often
101) I would comply as my community believes walking a dog is essential

102) I would comply with all the advice as my vet knows what is best for my dog's health

Scenario 4: Consider a hypothetical / imaginary scenario where your dog's veterinarian has advised that your dog's mental well-being would improve if you walked your dog more often and / or for a longer duration.

Please rate each of the following statements with regards to your response to this advice:

Likert Scale options for all: Strongly agree; Agree; Neither Agree nor Disagree; Disagree; Strongly Disagree; Not applicable

103) I would fully comply as my dog really enjoys going for walks
104) I would feel guilty if I could not walk my dog more often than I currently walk it
105) I feel that I would find it easier to meet my dog's mental well-being needs by taking her / him out for walks more often
106) I would enjoy the social interaction that I would have with other people on walks
107) I feel my family and friends would support this as they think it's important for me to take my dog for walks
108) I feel that it would be a difficult to modify my schedule to walk my dog more
109) I would comply as this is the way my dog goes to the toilet
110) I would comply as it's the time my dog gets outside
111) I would comply as that's the way my dog and I spend time together
112) I would worry that she / he would lose too much weight if I walked more often
113) I would comply as my community believes walking a dog is essential
114) I would comply with all the advice as my vet knows what is best for my dog's health

Thank you for taking the time to complete this survey.

Table S1: Results of Friedmans pairwise comparison tests for "I would comply with all the advice as my vet knows what is best for my dog's health" for walking more and less for behavioural and medical reasons:

Sample 1-Sample 2	Test Statistic	Std. Error	Std. Test Statistic	Sig.	Adj. Sig.	Interpretation
90. Vet Advice Walk Less for Behavioural Reason - 114. Vet Advice Walk More for Behavioural Reason	-0.383	0.055	-6.959	<0.001	0.000	Owners are more likely to comply with their vet's advice to walk their dog more for a behavioural reason than to comply with their vet's advice to walk less for a behavioural reason
90. Vet Advice Walk Less for Behavioural Reason - 102. Vet Advice Walk More for Medical Reason	-0.482	0.055	-8.752	<0.001	0.000	Owners are more likely to comply with their vet's advice to walk their dog more for a medical reason than to comply with their vet's advice to walk less for a behavioural reason.
90. Vet Advice Walk Less for Behavioural Reason -78. Vet Advice Walk Less for Medical Reason	0.659	0.055	11.959	<0.001	0.000	Owners are more likely to comply with their vet's advice to walk their dog less for a medical reason than to comply with their vet's advice to walk less for a behavioural reason
114. Vet Advice Walk More for Behavioural Reason - 102. Vet Advice Walk More for Medical Reason	0.099	0.055	1.793	0.073	0.437	Owners are just as likely to comply with their vet's advice to walk more for a behavioural reason as for a medical reason
114. Vet Advice Walk More for Behavioural Reason -78. Vet Advice Walk Less for Medical Reason	0.276	0.055	5.000	<0.001	0.000	Owners are more likely to comply with their vet's advice to walk their dog less for a medical reason than to comply with vet advice to walk more for a behavioural reason
102. Vet Advice Walk More for Medical Reason -78. Vet Advice Walk Less for Medical Reason	0.177	0.055	3.207	0.001	0.008	Owners are more likely to comply with their vet's advice to walk less for a medical reason than to comply with vet advice to walk more for a medical reason.

Each row tests the null hypothesis that the Sample 1 and sample 2 distributions are the same.
Asymptotic significances (2-sided tests) are displayed. The significance level is .050.
a. Significance values have been adjusted by the Bonferroni correction for multiple tests.

.

Table S2: Bivariate Analysis of medvsbehavpriority to determine where owners who prioritised medical over behavioural differed

Null hypothesis	p-value[a,b]	Decision	Interpretation
The distribution of “I do NOT feel that my vet expects me to walk my dog” is the same across categories of medvsbehavpriority.	<.001	Reject the null hypothesis.	Owners prioritise physical over behavioural health
The distribution of “I feel that my dog trainer expects me to walk my dog” is the same across categories of medvsbehavpriority	0.937	Retain the null hypothesis.	Owners did not prioritise physical over behavioural
The distribution of “Walking is integral to how my dog goes to the toilet” is the same across categories of medvsbehavpriority.	<.001	Reject the null hypothesis.	Owners prioritise physical over behavioural health
The distribution of “Walking my dog is an important part of my daily routine” is the same across categories of medvsbehavpriority.	<.001	Reject the null hypothesis.	Owners prioritise physical over behavioural health
The distribution of “Walking is the only time my dog gets outside” is the same across categories of medvsbehavpriority.	0.003	Reject the null hypothesis.	Owners prioritise physical over behavioural health
The distribution of “My dog’s behaviour has a big influence on how, when and/or where I walk” is the same across categories of medvsbehavpriority.	0.371	Retain the null hypothesis.	Owners did not prioritise physical over behavioural health
The distribution of “My dog’s physical health affects how, when and /or where I walk” is the same across categories of medvsbehavpriority.	0.494	Retain the null hypothesis.	Owners did not prioritise physical over behavioural health
The distribution of “My dog has access to an enclosed area (e.g. garden / dog park) and therefore I do NOT feel that my dog needs to go for walks” is the same across categories of medvsbehavpriority	0.121	Retain the null hypothesis.	Owners did not prioritise physical over behavioural health
The distribution of “Walking is important “quality time” my dog and I spend together” is the same across categories of medvsbehavpriority.	0.136	Retain the null hypothesis.	Owners did not prioritise physical over behavioural health
The distribution of “I have easy access to dog friendly walking tracks and paths for walking my dog” is the same across categories of medvsbehavpriority	0.038	Reject the null hypothesis.	Owners prioritise physical over behavioural health
The distribution of “I believe that my community is supportive of me walking my dog in the local neighbourhood” is the same across categories of medvsbehavpriority.	0.276	Retain the null hypothesis.	Owners did not prioritise physical over behavioural health
The distribution of “I feel that my dog is difficult to manage at home if I don’t take him / her for a walk” is the same across categories of medvsbehavpriority.	0.136	Retain the null hypothesis.	Owners did not prioritise physical over behavioural health
The distribution of “I feel that my dog gets excited to go for walks” is the same across categories of medvsbehavpriority.	0.024	Reject the null hypothesis.	Owners prioritise physical over behavioural health
The distribution of “I believe that walking my dog is important for my dog’s physical health” is the same across categories of medvsbehavpriority.	0.005	Reject the null hypothesis.	Owners prioritise physical over behavioural health
The distribution of “I feel that walking my dog is important for my own physical health” is the same across categories of medvsbehavpriority.	0.007	Reject the null hypothesis.	Owners prioritise physical over behavioural health
The distribution of “I feel that walking my dog is important for me to see friends / family / neighbours” is the same across categories of medvsbehavpriority.	<.001	Reject the null hypothesis	Owners prioritise physical over behavioural health
The distribution of “I feel that my family and/or friends think that it’s good that I walk the dog” is the same across categories of medvsbehavpriority	0.085	Retain the null hypothesis.	Owners did not prioritise physical over behavioural health
The distribution of “I feel that members of my family or a “significant other” thinks that walking a dog is the responsible thing to do as a dog owner” is the same across categories of medvsbehavpriority.	0.002	Reject the null hypothesis.	Owners prioritise physical over behavioural health
The distribution of “I feel that the community I live in expects dogs to be walked regularly” is the same across categories of medvsbehavpriority.	0.012	Reject the null hypothesis.	Owners prioritise physical over behavioural health

The distribution of "I feel that walking is important for my dog's mental well-being" is the same across categories of medvsbehavpriority	0.348	Retain the null hypothesis.	Owners did not prioritise physical over behavioural health
The distribution of "I feel that walking with my dog is important for my own mental well-being" is the same across categories of medvsbehavpriority.	0.032	Reject the null hypothesis.	Owners prioritise physical over behavioural health
The distribution of "I believe that walking is a relaxing activity for my dog" is the same across categories of medvsbehavpriority.	0.059	Retain the null hypothesis	Owners did not prioritise physical over behavioural health
The distribution of "Walking my dog is N0T a relaxing / enjoyable activity for me" is the same across categories of medvsbehavpriority.	0.021	Reject the null hypothesis.	Owners prioritise physical over behavioural health
The distribution of "I do NOT feel that walking my dog is important for my dog's social interactions with people" is the same across categories of medvsbehavpriority	<0.001	Reject the null hypothesis.	Owners prioritise physical over behavioural health
The distribution of "I believe that my dog keeps me safe when out walking" is the same across categories of medvsbehavpriority.	0.483	Retain the null hypothesis.	Owners did not prioritise physical over behavioural health
The distribution of "Walking my dog gives me important "quality time" in nature" is the same across categories of medvsbehavpriority	<0.001	Reject the null hypothesis.	Owners prioritise physical over behavioural health
The distribution of "Walking gives my dog important "quality time" in nature" is the same across categories of medvsbehavpriority.	<0.001	Reject the null hypothesis.	Owners prioritise physical over behavioural health
The distribution of "I feel that walking my dog is expected of me" as a responsible dog owner is the same across categories of medvsbehavpriority	0.003	Reject the null hypothesis.	Owners prioritise physical over behavioural health
The distribution of "I do N0T look forward to going for walks with my dog" is the same across categories of medvsbehavpriority.	0.020	Reject the null hypothesis.	Owners prioritise physical over behavioural health
The distribution of "Other people's complaints / criticisms have affected how, when and/or where I walk my dog" is the same across categories of medvsbehavpriority	0.486	Retain the null hypothesis	Owners did not prioritise physical over behavioural health
The distribution of "Going for walks near my home is unsafe, so I have to plan how, when and/or where to walk my dog" is the same across categories of medvsbehavpriority.	0.186	Retain the null hypothesis.	Owners did not prioritise physical over behavioural health
The distribution of "My work schedule makes it difficult to walk my dog" is the same across categories of medvsbehavpriority	0.327	Retain the null hypothesis.	Owners did not prioritise physical over behavioural health
The distribution of "I feel that family commitments make it difficult to walk my dog" is the same across categories of medvsbehavpriority.	0.021	Reject the null hypothesis.	Owners prioritise physical over behavioural health
The distribution of "I feel that one or more people in my community do not like dogs, so I find it difficult to walk near my home" is the same across categories of medvsbehavpriority	0.838	Retain the null hypothesis.	Owners did not prioritise physical over behavioural health
The distribution of "My friends / family say that my dog should N0T be walked" is the same across categories of medvsbehavpriority	0.278	Retain the null hypothesis	Owners did not prioritise physical over behavioural health
The distribution of "Walking is my dog's only exercise" is the same across categories of medvsbehavpriority.	0.403	Retain the null hypothesis.	Owners did not prioritise physical over behavioural health
The distribution of "My vet has advised that I should modify where / when / how I should walk my dog" is the same across categories of medvsbehavpriority	0.863	Retain the null hypothesis.	Owners did not prioritise physical over behavioural health
The distribution of "My dog trainer has advised that I should modify where / when / how I should walk my dog" is the same across categories of medvsbehavpriority.	0.880	Retain the null hypothesis.	Owners did not prioritise physical over behavioural health
The distribution of "I believe that walking my dog is important for my dog's social interactions with other dogs" is the same across categories of medvsbehavpriority	<.001	Reject the null hypothesis.	Owners prioritise physical over behavioural health

a. The significance level is 0.050.
b. Asymptotic significance is displayed.
